# Urinary Prognostic Biomarkers and Classification of IgA Nephropathy by High Resolution Mass Spectrometry Coupled with Liquid Chromatography

**DOI:** 10.1371/journal.pone.0080830

**Published:** 2013-12-05

**Authors:** Shiva Kalantari, Dorothea Rutishauser, Shiva Samavat, Mohsen Nafar, Leyla Mahmudieh, Mostafa Rezaei-Tavirani, Roman A. Zubarev

**Affiliations:** 1 Department of Basic Science, Faculty of Paramedical Sciences, Shahid Beheshti University of Medical Sciences, Tehran, Iran; 2 Department of Medical Biochemistry and Biophysics, Karolinska Institute, Stockholm, Sweden; 3 SciLifeLab, Stockholm, Sweden; 4 Department of Nephrology, Shahid Labbafinejad Medical Center, Shahid Beheshti University of Medical Sciences, Tehran, Iran; 5 Urology and Nephrology Research Center, Shahid Beheshti University of Medical Sciences, Tehran, Iran; 6 Proteomics Research Center, Faculty of Paramedical Sciences, Shahid Beheshti University of Medical Sciences, Tehran, Iran; Institut national de la santé et de la recherche médicale (INSERM), France

## Abstract

IgA nephropathy is the most common cause of primary glomerulonephritis. There are different pathologic biopsy-based scoring systems in use, but there is no consensus among nephrologists yet regarding the best classification method. Our aim was to test urine proteomics as a non-invasive method for classification of IgA nephropathy. This aim was pursued by discovering novel prognostic protein biomarkers in urine, and linking them to pathogenesis of the disease through known signaling and metabolic pathways. 13 urine samples of the patients with biopsy-proven IgA nephropathy were analyzed via two proteomics approaches: nanoflow LC-MS/MS and GeLC-MS/MS. The results of label-free quantification were subjected to multivariate statistical analysis, which could classify patients into two groups, broadly corresponding to the primary and advance stages. The proteome classification correlated well with biopsy-based scoring systems, especially endocapillary hypercellularity score of the Oxford’s classification. Differentially excreted candidate proteins were found as potential prognostic biomarkers: afamin, leucine-rich alpha-2-glycoprotein, ceruloplasmin, alpha-1-microgolbulin, hemopexin, apolipoprotein A-I, complement C3, vitamin D-binding protein, beta-2-microglobulin, and retinol-binding protein 4. Pathway analysis suggested impairment of Extra Cellular Matrix (ECM)-Receptor Interaction pathways as well as activation of complement and coagulation pathway in progression of IgA nephropathy.

## Introduction

IgA nephropathy is the most common cause of primary glomerulonephritis throughout most of the developed world [1]. Although previously thought to be a benign pathology, less than 10% of patients have complete remission and 6% to 43% of patients develop end-stage renal disease in 10 to 20 years after the initial diagnosis [1,2]. 

Many efforts have been made over decades of research to predict the clinical course of the disease and to design a prognostic scoring system based on demographic (age and gender), clinical (hypertension), laboratory (creatinine level and proteinuria at presentation), and pathologic indices [3,4]. Different pathologic scoring systems were developed over decades, notably the H.S. Lee’s glomerular grading system and the Oxford histologic classification of IgA nephropathy [5-8].

According to the H.S. Lee’s system, biopsy specimens are graded into five different groups: grade I, normal or focal mesangial cell proliferation; grade II, diffuse mesangial cell proliferation or <25% of glomeruli with crescent (Cr)/segmental sclerosis (SS)/global sclerosis (GS); grade III, 25–49% of glomeruli with Cr/SS/GS; grade IV, 50–75% of glomeruli with Cr/SS/GS; and grade V, >75% of glomeruli with Cr/SS/GS [6].

In 2009, an international consensus working group has developed Oxford classification which is based on four histiopathological features (MEST): mesangial hypercellularity (M), endocapillary hypercellularity (E), segmental glomerulosclerosis (S), and tubular atrophy and interstitial fibrosis (T) [7,8]. According to the Oxford classification, both chronic fibrotic changes and mesangial and endocapillary hypercellularity can provide the prognosis. Since then, several studies have been performed to validate the Oxford classification in different cohorts [9-12].

The results have been somewhat inconsistent [13]. These inconsistencies, the limitations in risk stratification of IgA nephropathy and the invasive nature of kidney biopsy bring the need for novel IgA nephropathy biomarkers into the spotlight.

The urine proteomics approach is a noninvasive and promising novel method for evaluating the changes in protein patterns as a potential prognostic marker for predicting the clinical course of IgA nephropathy [14]. Here we study the differences in urine proteome patterns of different pathological classifications of IgA nephropathy and their relation to clinical and pathological indices. The hope was to find a prognostic biomarker among urine proteins, and to learn more about the mechanisms involved in the pathogenesis of IgA nephropathy. The number of patients (13) was in line with recent studies on urine proteomes (e.g. in reference [15], seven subjects have been studied). The limited goal of our study (differentiation between stages of a known disease) and the fact that each subject provided not only urine sample but also kidney biopsy, justifies such a limited cohort. 

## Methods

### Patients

At Labbafinejad Hospital, thirteen patients (11 males and 2 females) with biopsy-proven IgA nephropathy were consecutively enrolled in this study during 2011. Age, sex, smoking habits and also diet (a day before sampling) of the patients were noted and patients with other implications like diabetes were excluded. None of the patients had gross hematuria at the time of sampling. Written consents were provided for participants according to recommended consent form of "Medical Ethics" committee of Shahid Beheshti University of Medical Sciences. These consents were included these issues: brief introduction of study, advantages, dangers, confidentiality of the identity of participants, a contact information for answer to questions, the right for resign of the study. Each participant signed the consent form because collecting urine samples were non-invasive and simple. "Medical Ethics" committee of Shahid Beheshti University of Medical Sciences approved this consent procedure and also approved this study. IgA nephropathy was histologically classified as class I–V, according to both H.S. Lee’s classifications and the Oxford classification (MEST). For each patient, data were collected concerning serum creatinine, eGFR (estimated glomerular filtration rate) (by CKD-EPI equation (chronic kidney disease epidemiology collaboration)), presence of hypertension, MAP (mean arterial blood pressure), and proteinuria at presentation. 

### Collection of urine samples and protein extraction

Approximately 20-40 mL of second morning midstream urine from patients were collected and 1 mL of dissolved protease inhibitor (one tablet, Cocktail protease inhibitor, Sigma, dissolved in distilled water) was added to each 10 mL of urine. The samples were centrifuged at 3000 rcf for 20 minutes at 4 °C to pellet the cell debris. The supernatant was transferred into 15 mL tubes and stored at -80 °C until the samples were processed further. The supernatant were concentrated and desalted by ultrafilteration as follow: urine samples were transferred to individual Amicon Ultra-15 Centrifugal Filter Units with a 3 kDa cutoff (Millipore, Billerica, MA, USA) and spun at 3220 rcf at 4°C for 1h. The initial concentration was followed by two wash steps by adding 14 mL of PBS and spinning each tube at 3220 rcf at 4 °C for 1 h. By filtering, the sample volume was reduced from 15 mL to a ﬁnal volume of approximately 800-1000 µL. In order to inactivate potential bacterial activity, 1200 µL of cold acetone was added to 300 µL of concentrated urine and incubated at -20 °C overnight. The samples were dried in a vacuum concentrator and stored at -20 °C.

### Sample preparation for LC-MS analysis

Dried samples were re-suspended in 0.1 M ammonium acetate (pH 5) and the protein concentrations of the samples were determined using the BCA (Bicinchoninic Acid) Protein Assay (Pierce, Thermo Scientific, USA). 10 µg urinary proteins from the individual samples were digested in duplicates using sequencing-grade trypsin (Promega, USA). The samples were digested by trypsin in a ratio of 1:50 (enzyme:protein) at 37 °C overnight after reduction and subsequent alkylation in turn by 20 mM DTT (dithiothreitol) and 66mM IAA (Iodoacetamide). The resulting peptides were desalted using C18 StageTip (Thermo Scientific, USA) [16]. The eluted peptides were evaporated in a SpeedVac and re-suspended in a buffer containing 0.1% formic acid and 3% ACN (acetonitrile) v/v before loading to a nano-LC-MS/MS system. 

### Liquid chromatography tandem mass spectrometry

Liquid chromatography tandem mass spectrometry (nLC-MS/MS) analyses were performed on an Easy-nLC system coupled online to a Q Exactive mass spectrometer (both - Thermo Scientific, Bremen, Germany) Separation of peptides was performed using a 10 cm fused silica tip column (SilicaTips™ New Objective Inc., Woburn MA, USA) in-house packed with Reprosil-Pur C18-AQ 3 µm resin (Dr. Maisch GmbH, Ammerbuch-Entringen, Germany) using a methanol slurry and a pressurized ‘‘packing bomb’’ operated at 40 bar (Proxeon Biosystems). Mobile phases consisted of 0.1% formic acid in water v/v (buffer A) and 0.1% formic acid in acetonitrile v/v (buffer B). The LC gradient was set up as following: 5−35% buffer B in 89 min, 48−80% buffer B in 5 min, and 80% buffer B for 8 min, all at a flow rate of 300 nL/min. Samples (10 µL corresponding to approximately 2.3 µg of total protein) were injected via a temperature-controlled autosampler. 

The MS acquisition method was comprised of one survey full scan ranging from m/z 300 to m/z 1650 acquired with a resolution of R= 70,000 at m/z 400, followed by data-dependent HCD (high energy collision dissociation) MS/MS of maximum ten most abundant precursor ions with a charge state ≥ 2. MS/MS spectra were acquired with a resolution of R=17,500, with a target value of 2e5 ions, isolation m/z width was set to 4 and normalized collision energy to 26 eV. For all sequencing events, dynamic exclusion was enabled and unassigned charge states were rejected. 

### One-dimensional SDS-PAGE, in-gel digestion and cleaning procedure

Proteins (5 µg for each sample) were loaded on a 4-12% Bis-Tris mini gel (Novex, Invitrogen, Carlsbad, CA, USA) using 2-(N-morpholino)-ethanesulfonic acid or 3-(N-morpholino)-propanesulphonic acid SDS running buffer (Invitrogen), in accordance with manufacturer's instructions. After staining with Coomassie blue, each gel lane was cut into eight pieces and subjected to in-gel tryptic digestion. The digestion was performed by a liquid-handling robot (MultiProbe II, Perkin Elmer), including protein reduction in 10 mM DTT and alkylation in 55 mM IAA. Gel pieces were dehydrated in 100% acetonitrile, trypsin was added to a final concentration of 13 ng/µL, and the pieces were digested for 5 h at 37 °C. Extracted peptides from consecutive bands were pooled according to their protein levels, resulting in eight pools for each lane.

The extracts were concentrated in vacuum and then de-salted using C18 ZipTips(Millipore, Billerica, MA, USA,). ZipTips were first wetted with 100% acetonitrile and then equilibrated with a solution containing 3% acetonitrile and 0.1% TFA (trifluoroacetic acid). Elution of the peptides was performed with 80% acetonitrile and 0.1% TFA. Dried peptides were solubilized again in a 0.1% formic acid in water (HPLC grade) prior to loading onto the nano-LC column. 

### MS data analysis

Tandem mass spectra were extracted using Raw2MGF (in-house-written program) and the resulting Mascot generic files (.mgf) were searched against a concatenated SwissProt protein database (Human taxonomy) using Mascot 2.3.0 search engine (Matrix Science Ltd., London, UK). Carbamidomethylations of cysteins was set as a ﬁxed modiﬁcation and deamidation of asparagine and glutamine as well as oxidation of methionine were set as variable modiﬁcations. Up to two missed tryptic cleavages were allowed and the mass tolerance was set to 10 ppm and to 0.05 Da for the precursor and fragment ions, respectively. Only peptides having individual MS/MS Mascot score above signiﬁcant threshold corresponding to E<0.05 were accepted. Only proteins identified with at least two peptides with a significant score and at 0.25% false discovery rate (FDR) were considered for further quantification. 

Relative abundance of proteins identified with ≥ 2 unique peptides and a significance threshold of E < 0.05 was determined using Quanti (an in-house developed software package) [17]. The areas of the chromatographic peaks were taken as the peptide abundances and the same peptides were quantiﬁed in each nLC-MS/MS data file using accurate mass and the order of elution as identifiers. The sum of the abundances of all unique peptides of a protein was used as the protein abundance value. The list of quantified proteins was further filtered to 1% FDR, which corresponded to the protein Mascot score of 23.63 for in solution digestion and 28.76 for in gel digestion. 

### Statistical analysis

The quantitative proteome data was subjected to multivariate statistical analysis using SIMCA (SIMCA-p 13.0, Umetrics, Umeå, Sweden). Unsupervised principal component analysis (PCA) [18,19] was performed without consideration of group information. By reducing the number of proteins in the PCA input based on the number of peptides per protein (by keeping the proteins with larger number of peptides, i.e. most reliable data), several PCA were performed. In order to find the best model with a maximal distance between the groups, a quality factor was calculated for each model using the following empirical formula:

Q=IA_(x,y)_-B_(x,y_)I^2^/(δ^2^A_x_+δ^2^B_x_+δ^2^A_y_+δ^2^B_y_),

where A_x_ is the average x value (from PC1) for all samples with advanced stage of the disease (class IV and V according to H.S. Lee’s classification), B_x_ is the average x value for all samples with primary stage of the disease (class II and III according to H.S. Lee’s classification), A_y_ and B_y_ are the average y values (from PC2) for patients in advanced and primary stages, respectively, and δ^2^A_x_, δ^2^B_x_, δ^2^A_y_, δ^2^B_y_ represent the standard deviations of x and y values for the samples in two stages.

The model with the highest quality factor was selected as the best model, and significant (according to PCA) proteins were considered as potential biomarkers. 

PLS (Partial Least Squares) analysis was applied using SIMCA in order to introduce a predictive model for stage classification of IgA nephropathy based on proteomics data obtained by PCA. In PLS, the X matrix contains the data variables (all quantified proteins), while the Y matrix contains the class variable for which values are chosen to be the class descriptor (predictive biomarkers which were common in GeLC-MS/MS and nLC-MS/MS) [20]. Unlike other popular dimension reduction techniques, such as principal components analysis, the PLS algorithm calculates each latent variable from X based on Y. The objective is to maximize the covariance between Y and X, unlike PCA which maximizes the variance of the variables, X, alone. Thus PLS, unlike PCA, explicitly accounts for the covariates within the model [21].

### Protein GO-term Enrichment, Pathway and Regulator Analysis

Gene ontology annotation was performed for the proteins in the data set, pathways and regulator analysis was done using the “GeneXplain platform” (GenExplain GmbH, Wolfenbüttel, Germany) and DAVID open-source software tool [22]. DAVID uses the EASE score [23], a modiﬁed Fisher Exact p-value, to determine whether a GO-term is over- or under-represented in a given proteomic data set with reference to a background data set (e.g. the human proteome). To identify the upstream key nodes of proteins of interest, regulator analysis was performed using a”GeneXplain” tool with a FDR of 0.05 and the TRANSPATH database. Key nodes are signaling molecules found on pathway intersections in the upstream vicinity of the genes from the input list. Each key node was given a score reflecting its connectivity, i.e. how many input-list genes were reached and the proximities to those genes. The score calculation also included the abundances of the downstream proteins detected in the proteomics experiment [24].

## Results

### Clinical and pathological characteristics of patients

Clinical and laboratory information on patients are provided in Table 1. Thirteen patients (11 males and 2 females, between 18-52 years old, mean age 33 years) with biopsy-proven IgA nephropathy were enrolled. The biopsy samples were classified by a single pathologist according to both H.S. Lee’s and Oxford classifications. In order to estimate the amount of protein excretion, a 24 h urine collection was used. The mean 24 h protein concentration was 3010 mg/day, with ten patients having urine protein concentration higher than 1 g/day. Renal function was evaluated by eGFR using CKD-EPI equation. The mean eGFR level was 67.7 cc/min/1.73 m^2^ and six patients had an eGFR of less than 60 cc/min/1.73 m^2^. Six patients had been diagnosed with hypertension in the past and were on antihypertensive treatments at presentation. 

**Table 1:** Demographic and laboratory characteristic of patients with IgA nephropathy.

Oxford Classification	H.S.Lee’s Classification	Proteinuria (mg/day)	eGFR (cc/min/1.73m^2^)	Sex	Age (yr)	Case
M1 S1 E0 T0	II	4600	44.10	M	52	1
M1 S0 E0 T1	III	1000	119.42	M	18	2
M1 S0 E1 T2	V	6000	8.58	M	29	3
M1 S0 E0 T0	II	6420	79.52	M	42	4
M1 S1 E1 T2	V	7020	46.60	M	29	5
M0 S1 E0 T0	III	1680	117.91	F	28	6
M1 S1 E0 T1	IV	4100	49.04	M	32	7
M1 S0 E1 T2	V	2330	16.11	M	28	8
M1 S1 E1 T1	III	800	63.65	F	23	9
M1 S0 E0 T0	II-III	1310	97.76	M	34	10
M1 S1 E1 T0	III	720	68.01	M	45	11
M1 S1 E0 T1	V	2640	35.71	M	34	12
M1 S0 E0 T0	II-III	520	133.51	M	42	13

(eGFR: Estimated Glomerular Filtration Rate by CKD-EPI equation; Oxford Classification M: Mesensial hypercellularity, S: Segmental glomerulosclerosis, E: Endocapilary hypercellularity, T: tubular atrophy/ interstitial fibrosis).

### Principal Component Analysis (PCA) of in-solution digestion proteomes

**Figure 1:**
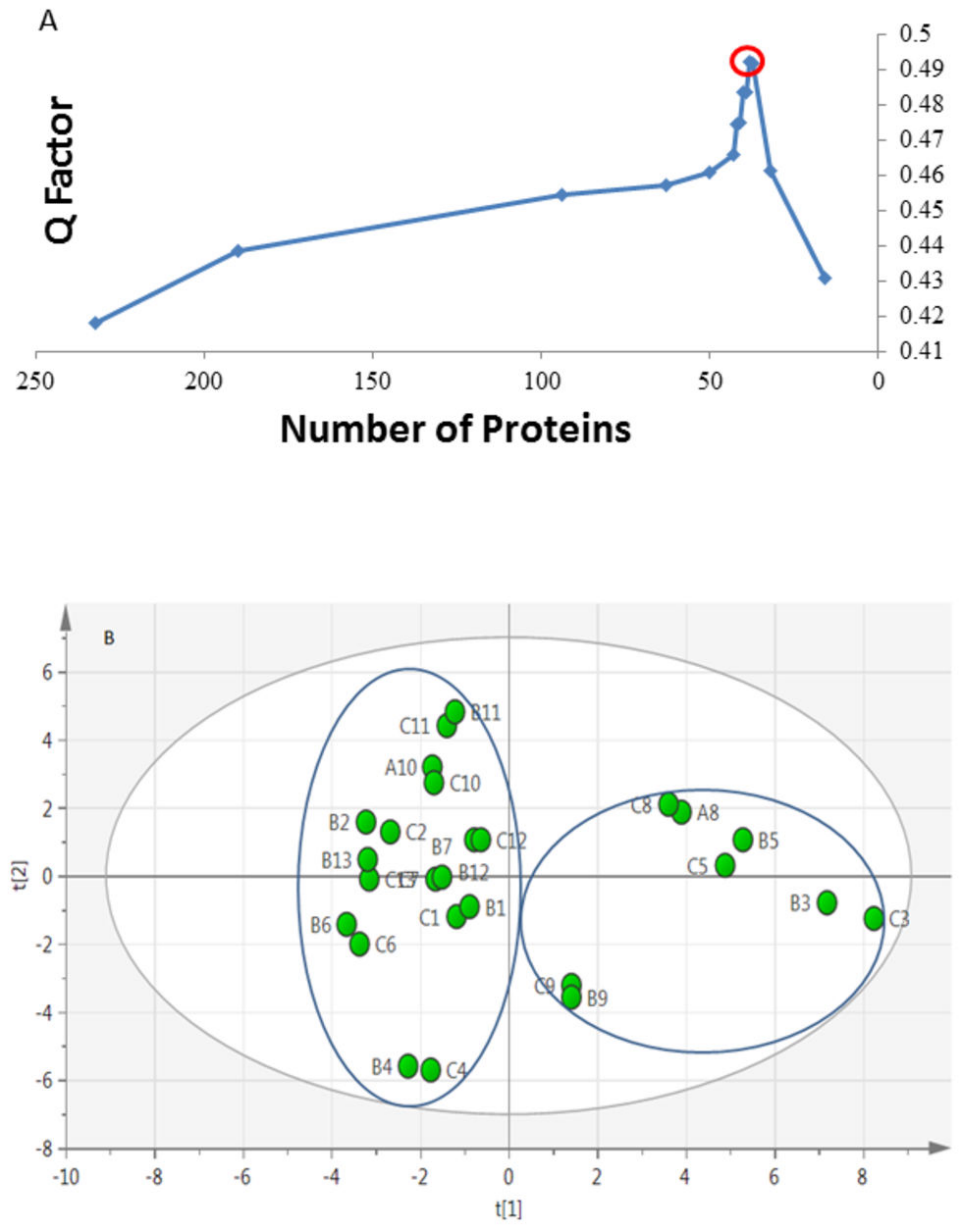
Quality factor Q against number of proteins N included in the model. The proteins were sorted according to the number of detected peptides per protein, and N top proteins were retained for the model. The highest Q corresponds to a model N=38 proteins, which was accepted as the best model (A). Unsupervised PCA scores plot based on 38 quantified proteins across all 13 samples. All the replicates were close to each other and there was no statistical outlier (B).

A total number of 232 unique proteins were identified and quantified by nLC-MS/MS. The proteins were sorted according to their relative abundances. Top N proteins (N was varied) were used for PCA; the optimal N=38 was selected (see Material and Methods) based on the best separation between the high and low disease stage groups (Figure 1A). Score plot (Figure 1B) shows two clusters separated along the main principal coordinate, PC1. Out of the 38 proteins used for classification, 18 proteins were with statistically significant abundance changes, and thus were identified to be the most important markers responsible for the observed clustering (Table S1). 

By and large, the obtained clustering of the proteomics data agrees with the biopsy results. In the first cluster, patients 1, 2, 4, 6, 7, 10, 11 and 13 are all in pathologic class II and III according to H.S. Lee’s classification and thus belong to the low stage group. In the second cluster, patients 3, 5 and 8 are all in pathologic class V and therefore belong to the high stage group. Patient 9, according to biopsy results, belongs to stage III, i.e. to the low stage, but the proteomics data are closer to the high-stage cluster. Another disagreement concerns the patients 7 and 12, who were in the high stage group according to biopsy, but whose proteomics results clustered together with the low stage group. 

In attempt to verify the proteomics-based classification, 1D GeLC-MS/MS analysis was performed. In this approach, instead of being directly digested in solution, the urine proteins were first separated on a 1D gel, with each lane being cut in eight pieces, individually digested and run by nLC-MS/MS. In-solution and in-gel digestion often produce somewhat complementary results in terms of peptide abundances, but both types of analyses reflect the same biological differences between the samples. Since gel-based analysis takes more work and time to perform than in-solution digestion, it is less suitable in clinical setting, and thus we used it here just for verification. 

### PCA of in-gel-digested proteomes

**Figure 2:**
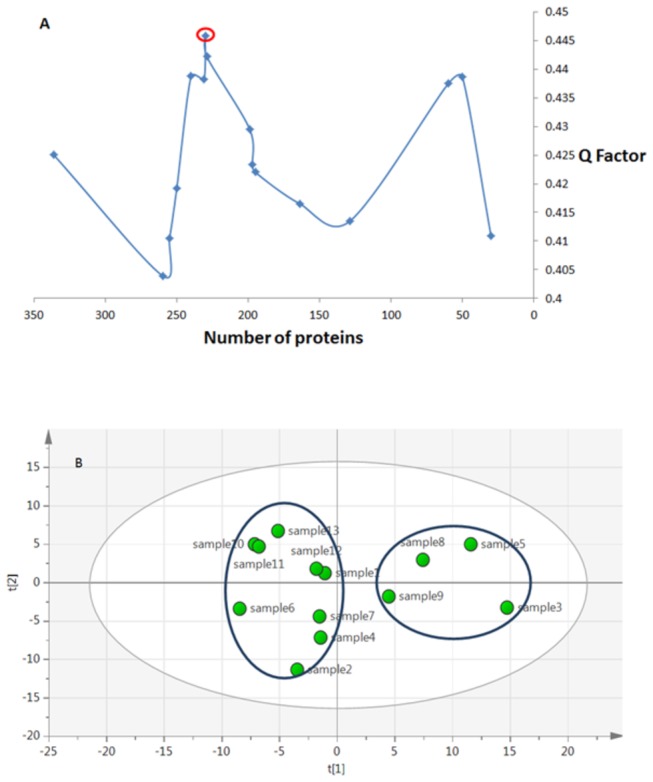
Quality factor plot for 1D GeLC-MS/MS. The best model corresponded to 230 proteins (A). Score plot of PCA using 1D GeLC-MS/MS (B).

A total of 336 unique proteins were detected and quantified by 1D GeLC-MS/MS. The larger number of proteins compared to in-solution digestion was not surprising, because each sample produced eight separately analyzed fractions. PCA with N top proteins (Figure 2A) gave the best model at N=230 (Figure 2B). The results generated by this method were consistent with those of nLC-MS/MS. Again, two main clusters were observed, separated along the main coordinate PC1. Patient 9 data again clustered together with the high stage group, while patients 7 and 12 belonged to the low stage cluster, contrary to biopsy results but in agreement with in-solution analysis. Note also that the order of disease severity in the high-stage patients, according to both analyses, was 9<8<5<3. Such conformity between the two proteomics approaches testifies to the high precision of proteomics measurements. 

Seventy three out of 230 proteins had significant abundance changes between the disease stages, and were the most important proteins responsible for the best clustering. After applying an additional filter criterion, where only proteins with a fold change of 1.5 or higher were retained, 62 proteins remained, of which 31 proteins were upregulated (overrepresented) in higher disease stages and 31 proteins were downregulated, or under-represented (Table S2).

All significant proteins obtained in either GeLC-MS/MS (61 proteins) or nLC-MS/MS (18 proteins) were used in GO and pathway analyses.

### Molecular weight analysis

In general, glomerular permeability is affected by molecular weight of proteins, as well as their size, shape and electrical charge. Normally, the renal threshold is ca. 68 kDa, therefore the glomerular filtrate contains proteins with lower molecular weight (MW) than the threshold. These filtered proteins are then taken up and catabolized by renal tubular epithelial cells, therefore in disease, these proteins can appear in the urine. Glomeruli malfunction may also result in high-MW proteins in urine. Thus the most straightforward hypothesis is that of the link between the protein MW and the disease stage. This hypothesis has been considered in some studies [25,26] which implied tubular damage and diminished protein reabsorption in proximal tubule as an upstream reason for excretion of low molecular weight proteins (B2MG, AMBP). To test this hypothesis in our data, we have analyzed the MW distribution of the significant proteins. Seven out of eight (87%) proteins reported as over-represented biomarkers in advanced stage are found to be below the physiological threshold of glomeruli, and have mean MW of 37 kDa. The MW of these low molecular weight (LMW) proteins (RET4, B2MG, APOA4, VTDB, APOA1, HEMO, AMBP) were significantly different from the under-represented proteins (p=0.048). This finding is consistent with the suggestion that evaluation of LMW proteins could be an indication for severity of glomerular disease such as IgA nephropathy. However, our proteomics results are not limited to the above finding. 

### Proteomics-based predictive model

**Figure 3:**
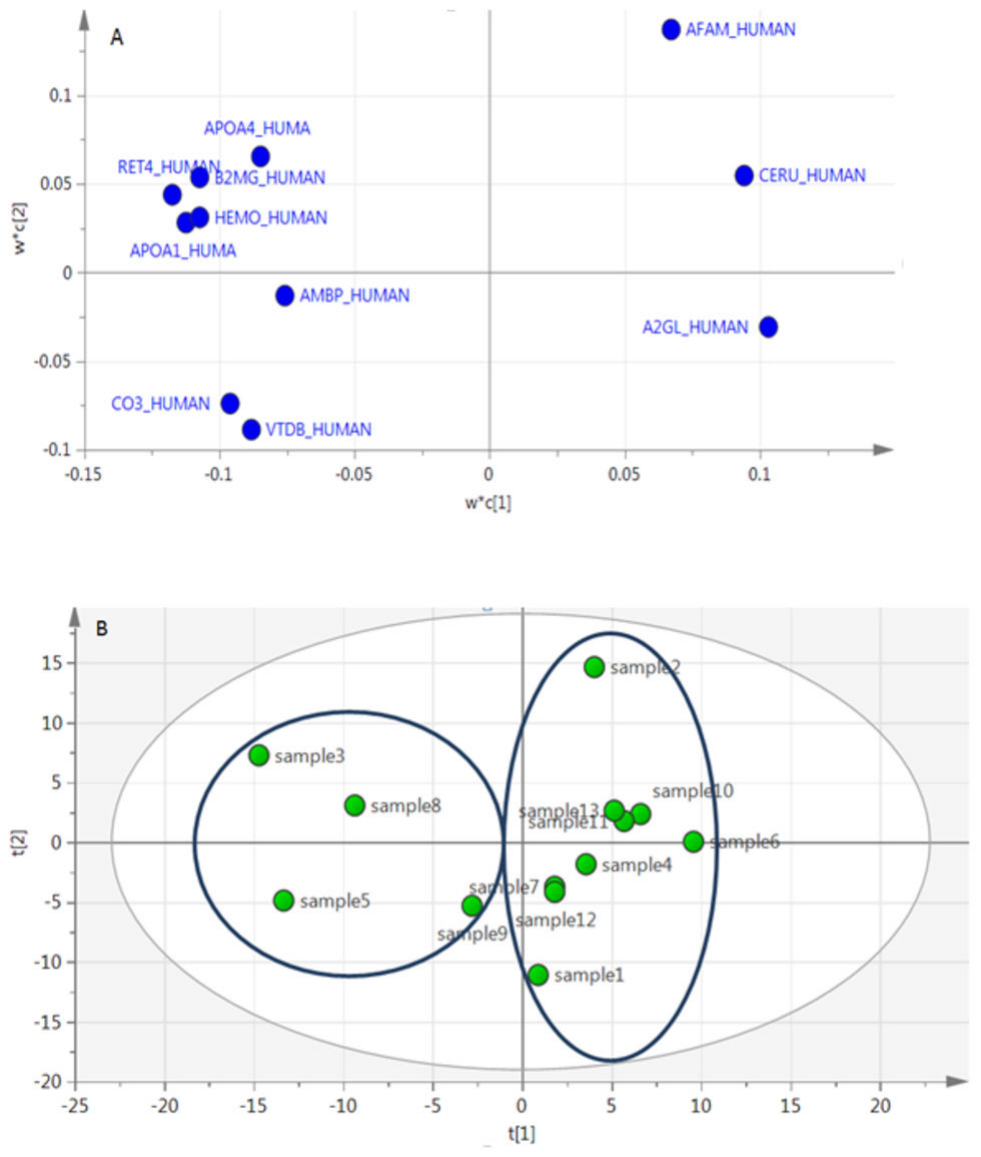
Proteomics-based predictive model using PLS analysis based on loading plot. The variables on the right are under-represented proteins and the variables on the left are over-represented proteins in advanced stage of IgA nephropathy (A). Score plot of Predictive model (B).

Owing to the significant proteins obtained from PCA results of in-solution digestion and in-gel digestion proteomes, a predictive model could be built for distinguish between primary and advanced stage of IgA nephropathy (Figures 3A and 3B). This model was constructed using PLS (Partial Least Squares) method [27]. The plot of X and Y loading weights (w* and c) of PLS component 1 against component 2 shows how the X-variables correlate with Y- variables, where X- variables are quantified proteins and Y-variables are prognostic biomarkers common in the best PCA models obtained from both proteomics methods (Table 2). For better clarity, only the Y-variables are shown on the Figures 3A and 3B. The proteins on the right are under-represented and the proteins on the left are over-represented in the advanced stages. The predictive (Q^2^) and fitness values (R^2^) were 0.64 and 0.864, respectively, which means high quality of the model. 

**Table 2:** A new classification based on proteomics data and comparison with other classification based on biopsy.

Patients code based on PC1	Proteomics-based classification (stage)	H.S. Lee’s Classification (stage)	Oxford Classification based on E score (stage)
3	advanced	advanced	advanced
5	advanced	advanced	advanced
8	advanced	advanced	advanced
9	advanced	primary	advanced
1	primary	primary	primary
2	primary	primary	primary
4	primary	primary	primary
6	primary	primary	primary
7	primary	advanced	primary
10	primary	primary	primary
11	primary	primary	advanced
12	primary	advanced	primary
13	primary	primary	primary

## Discussion

We have performed label-free proteomics analyses of urine samples from patients with IgA nephropathy using nLC-MS/MS and GeLC-MS/MS approaches. The obtained quantitative data were subjected to a multivariate analysis in order to classify patients based on the disease severity as well as finding novel diagnostic biomarkers. 

### Classification based on proteomic results

Based on the clustering of PCA analysis of nLC-MS/MS and 1D GeLC-MS/MS data sets, a classification model for IgA patients could be built which demonstrates 60% sensitivity and 87% specificity in comparison with H.S. Lee’s classification and 80% sensitivity and 100% specificity in comparison with Oxford classification (see the comparison in Table 2). Thus the proteomic results were more consistent with Oxford than H.S. Lee’s classification. For patients 9 and 11 who were classified as stage III by H.S. Lee’s classification but had endocapillary proliferation and therefore were E1 in Oxford classification which is associated with worse prognosis, proteomic data clustered together with patients who had more severe disease (stages IV and V). 

There is still disagreement on the relevance of the pathologic variable E (endocapillary hypercellularity) of the Oxford classification to the severity and prognosis of IgA nephropathy [13]. Based on urine protein profiles, patients with high stage of IgA nephropathy (samples number 3, 5, 8 and 9) correspond to E1 variable in the Oxford classification, while patients with low stage of the disease (except for patient 11) correspond to E0 (see Table 1 and Table 2). Since E1 is associated with worse prognosis than E0, the broad agreement between protein clustering and endocapillary proliferation suggests that the latter might be a better reflection of severity of IgA nepheropathy than the other variables (M, S and T). 

### Proteins Differentiating Pathologic Severity of IgA nephropathy

Proteins that were common in both types of proteomics analyses and which correlated with eGFR (Estimated Glomerular Filtration Rate; a parameter used evaluation of renal function) were selected as probable biomarkers for severity stratification (Table 3). These proteins were used as variables in our predictive model (Figure 3A and 3B). Association of all reported biomarkers in Table 3 were examined with proteinuria level which resulted in significant association of three proteins: APOA1 (p= 0.0239), APOA4 (p=0.0285) and CO3 (p=0.00865).

**Table 3:** Comparison of the proteomics-determined regulation of proteins and the values of eGFR (Estimated Glomerular Filtration Rate, an estimate of renal function).

p-value of correlation	Correlation with eGFR	Up/Down Regulation	Fold Change (GeLC-MS)	Fold change (LC-MS)	Biological process	Protein Name	Protein ID
0.001	0.634	↓	1.8	1.7	Vitamin ETransport	Afamin(Alpha-albumin)	AFAM
0.000	0.675	↓	2	1.7	Brown fat cell differentiation	Leucine-rich alpha-2-glycoprotein	A2GL1
0.000	0.666	↓	1.7	1.6	Ion Transport	Ceruloplasmin	CERU
0.001	-0.631	↑	1.9	2.6	cell adhesion	Alpha-1-microgolbulin	AMBP
0.002	-0.579	↑	2.7	6	Positive regulation of immunoglobulin production	Hemopexin	HEMO
0.003	-0.555	↑	5.8	7.2	Steroid metabolism	Apolipoprotein A-I	APOA1
0.001	-0.621	↑	1.7	8.2	Inflammatory response	Complement C3	CO3
0.004	-0.549	↑	5.3	11.1	Response to estradiol stimulus	Vitamin D-binding protein	VTDB
0.006	-0.527	↑	2.6	11.7	Positive regulation of fatty acid biosynthetic process	Apolipoprotein A-IV	APOA4
0.001	-0.593	↑	31	16.5	Regulation of immune response	Beta-2-microglobulin	B2MG
0.001	-0.616	↑	52	35.3	Positive regulation of immunoglobulin secretion	Retinol-binding protein 4	RET4

RET 4 (retinol binding protein 4) was reported by Li et al. [28] as a diagnostic biomarker for IgA nephropathy and the increased amount of it was positively related to tubulointerstitial lesion. The involvement of this LMW protein in regulation of immunoglobulin secretion (Inferred from sequence or structural similarity in GO database) might be the cause of its positive correlation with IgA nephropathy progression. 

Excessive losses of AMBP and B2MG have been known to be a sign of tubular damage since early proteomic studies of IgA nephropathy [29,30]. One of the molecular functions of AMBP is IgA binding [31], but the role of this protein in pathogenesis of IgA nephropathy is still unknown. Lundsberg et al. [32] studied blood samples of IgA nephropathy patients and implied the role of apolipoproteins as a risk factor for IgA nephropathy. However, Wang et al. [33] claimed that urinary Lp (a) excretion in various nephropathy patients (such as IgA nephropathy patients) was decreased compared to controls, while our results showed a clearly increased level of Apo AI and Apo AIV in advanced stage (p<0.0001 and p<0.0003, respectively) (Table 3). Downregulation of urinary ApoAI has been reported in diabetic patients with macroalbuminuria and was associated with disease progression [34]; however, Julian et al. reported ApoAI as a urinary biomarker of IgA nephropathy [35]. Consisted with that earlier finding, our data indicate upregulated urinary ApoAI in patients with more advanced disease.

ApoAIV, a glycoprotein with a known role in reverse transport of cholesterol, has recently been reported as a biomarker for prediction of progressive chronic kidney disease [36,37]. In our study, elevated urinary level of ApoAIV was also associated with advanced disease. ApoAIV is freely filtered by glomerulus and mostly reabsorbed by proximal tubule cells. Its plasma level correlates with chronic kidney disease progression [38]. Increased urinary excretion of ApoAIV is related to tubular injury and decreased reabsorption. Patients with tubular damage have increased urinary excretion of ApoAIV and AMBP [39]. As patients with more advanced IgA nephropathy are believed to be with more severe tubulointerstitial injury, this may explain the higher urinary level of ApoAIV.

The role of CO3 as a common key factor of three complement pathways (classical, lectin and alternative pathways) and its implication in pathogenesis of IgA nephropathy has been widely discussed [40,41]. A positive correlation between excessive loss of CO3 and advance stage of the disease was also observed in our results, but the main cause for the elevated amount of this protein and other complement pathway proteins in urine could not be clearly identified. 

In this study, we report for the first time an increased urinary excretion of VTDB (Vitamin D binding protein), A2GL (Leucin-rich alpha-2-glycoprotein), AFAM (Afamin) and HEMO (Hemopexin) in patients with more severe IgA nephropathy, and suggest these proteins to be predictive biomarkers for severity of IgA nephropathy.

Glycoproteins have critical role in cell-to-cell interaction and their urinary excretion may be an early marker of injury. Vivekanandan-Giri et al. reported altered urinary glycoprotein profile in CKD. Afamin, Hemopexin and leucin-riched alpha-2-glycoprotein were among those glycoproteins with altered expression in CKD, although their significance could not be shown [42].

Afamin (a-albumin, a1T-glycoprotein) is the newest member of the albumin family comprising albumin, a-fetoprotein, and vitamin D binding protein. Afamin mRNA expression is predominantly in liver and kidney [43].

Since here we are attempting not to diagnose IgA, but only to determine its severity, the found biomarkers do not have to have absolute specificity (i.e., be unique for IgA compared to other immune-mediated glomerulonephritis). Indeed, some of the potential biomarkers of the IgA nephropathy severity reported in the present study have previously been reported for other immune-mediated glomerulonephritis, although urinary proteomic studies are limited for such diseases. Proteins CERU [44], CO3 [45], A2GL1, HEMO, RET4, AMBP [46] and B2MG [47] have been found among urinary biomarkers for lupus nephritis and membranous glomerulonephritis. 

In addition, CO3 has been reported as tissue biomarker for immunotactoid glomerulopathy [48] and C3 Glomerulonephritis [49]. On the other hand, none of the reported in this study potential biomarkers have been associated with post-infectious glomerulonephritis or post-streptococcal glomerulonephritis. Moreover, to the best of our knowledge AFAM, VTDB, APOA-I and APOA-IV have not been associated with any other immune-mediated glomerulonephritis, and may be specific for IgA nephropathy.

### Gene Ontology enrichment, pathway and regulator analysis

Although urine itself does not support any metabolic or signaling pathway, it is instructive to perform GO classification of the significantly changing proteins and pathway analysis based on them, to obtain additional insight on the possible biological role of these proteins in IgA nephropathy.

**Figure 4:**
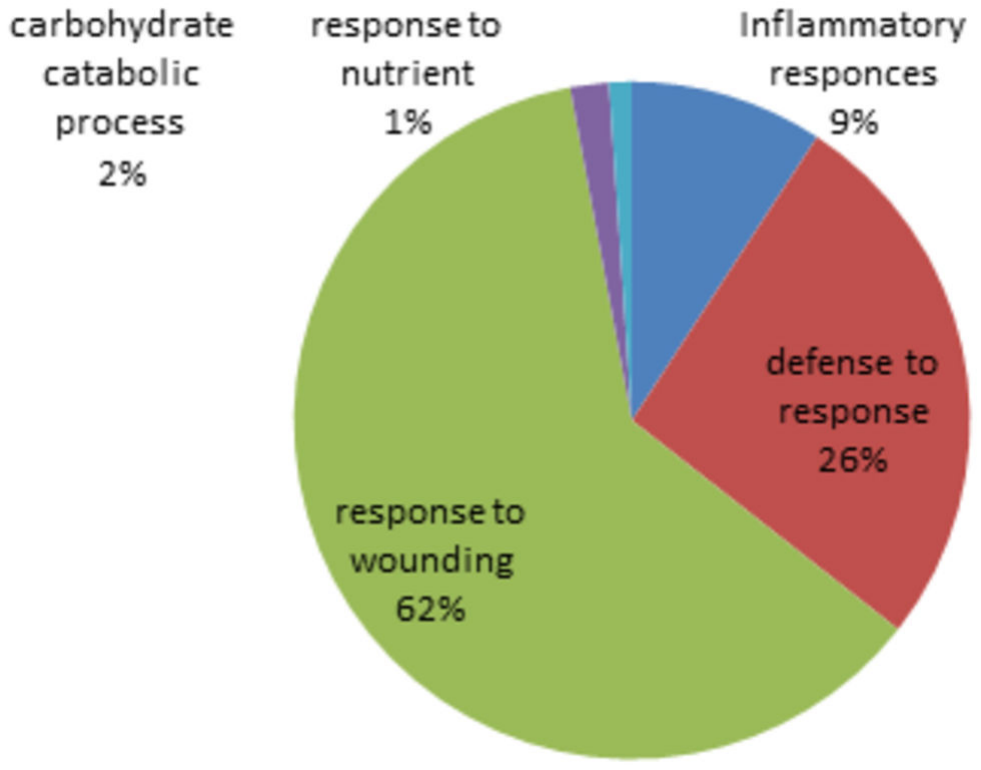
Gene Set Enrichment Analysis of biological Process. This analysis was done by "DAVID" using under-represented proteins obtained from both methods.

DAVID GO analysis yielded "response to wounding" as the most significant biological process (p=6.0x10-4) (Figure 4) for under-represented proteins and the only significant biological process present according to GeneXplain was lipid catabolic process (p=0.011). The significant cellular components and molecular function are listed in Table S3 and S4. Pathway analysis using DAVID with the KEGG database showed two major pathways, “Extra Cellular Membrane (ECM)-receptor interaction pathway” (p=0.02) and “lysosome” (p=0.0003) (Table S5). Upstream regulator and pathogenesis analyses were performed using GeneXplain (Table S6). The majority of master molecules (regulators) refer to CD44. 

According to DAVID, the most significant biological process (p=2×10^-5^) for over-represented proteins was “acute inflammatory response” (Table S7). Details of cellular component and molecular function for over-represented proteins have been tabulated at Table S8 and S9. Pathway analysis using the KEGG database revealed that the complement and coagulation pathway is the only significant (p=3×10^-6^) pathway. The results of regulator analysis are given in Table S10.

CD44 is present in four of the biological processes obtained from DAVID, pointing out the importance of this protein in progression of IgA nephropathy. Increased tissue expression of CD44 has been found in renal biopsy of IgA nephropathy, but Qiaoling et al. have found its expression to be significantly lower in the pathologic stage IV than III, which might be explained by the presence of more severe fibrosis [50]. Our analysis also demonstrates decreased urinary excretion of CD44 in more advanced stages of IgA nephropathy. Enrichment of this molecule in the GO-term, pathway and regulator analyses hints on its role in IgA nephropathy that can be further investigated.

**Figure 5:**
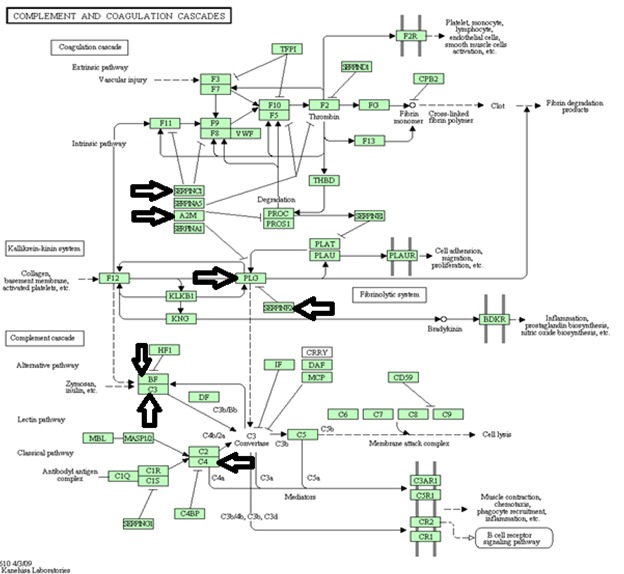
Complement-coagulation pathway from KEGG database [57, 58]. The proteins over-represented in our data set are marked by an arrow.

Activation of the Complement and coagulation pathway has been implied in a number of studies as one of the mechanisms of IgA nephropathy [40]. We suggest that over-representation of antithrombin-III (SERPINC1) andalpha-2-macroglobulin (A2M) as two major regulatory proteins of the coagulation pathway which observed in the dataset may explain implementation of complement and coagulation pathway in IgA nephropathy mechanism by inhibition coagulation cascade and thus leading to availability of substrates for the kallikerein-kinin system and complement cascade (Figure 5). 

Regulator analysis agreed with some previous findings and suggested a probable mechanism for progression of IgA nephropathy. Myeloperoxidase (MPO) is the master key of apolipoprotein a-1 (APOA1) that binds to high density lipoprotein (HDL) by APOA1 moiety. Oxidative modification by MPO of APOA1 in HDL can modify HDL and affect the cholesterol efflux from peripheral tissues to liver. By another mechanism, it can alter anti-inflammatory properties of HDL and convert it to a pro-inflammatory molecule [51]. Modified HDL by MPO can activate signaling cascade of NF-kB [50] that is believed to have important role in making atherosclerotic plaques. In our case, this can happen in kidney arteries and lead to inflammation and kidney injury.

Renin, another key node which is upstream of angiotensin in a number of known pathways, could play a role in IgA nephropathy progression by activation the intra-renal oxygen reactive species [52]. ADAM19 (Meltrin-beta) is the upstream regulator for alpha-2-macrogulbulin (A2M) whose role in renal disease has been identified in several studies [53]. The role of ADAM19 in progression of IgA nephropathy might be the regulation of the A2M downstream processes, such as binding to cytokines [54] which result in inflammation, or regulation of complement and coagulation pathway [55] by cleavage of this protein [56].

## Conclusions

In this study, we performed a classification of IgA nephropathy based on the results of two proteomic analyses based on semi-complementary nLC-MS/MS and GeLC-MS/MS approaches. Classification based on protein pattern was broadly consistent with Oxford’s classification and supported the importance of endocapilary hypercellularity as a prognostic feature. Eleven protein candidates identified with both methods were found to be the most probably prognostic biomarkers. A correlation was confirmed between excretion of LMW proteins and advanced disease stages. A proteomics-based predictive model was built that showed 80% sensitivity and 100% specificity in comparison with Oxford classification. Complement and coagulation pathways as well as Extra Cellular Membrane (ECM)-receptor interaction pathway were found as the most probable pathways in progression of IgA nephropathy. Using regulator analysis, myeloperoxidase, renin and ADAM19 were identified as important probable players in disease pathogenesis also candidates for future targeted experiments. 

Most proteins identified as potential biomarkers in this work have been implicated in other diseases as well. This however is not diminishing the practical utility of the findings, as they are to serve a limited purpose of differentiation between the stages of a known disease. 

Overall, this study found urine proteomics to be an informative and noninvasive method for determining the severity of IgA nephropathy. Since the disease classification based on the invasive kidney biopsy does not always correctly provide prognosis and predict response to treatment, proteomics can be a helpful addition to, and with time could become even a replacement of, the established invasive diagnostic approaches.

## Supporting Information

Table S1 **The most important 18 markers responsible for the clustering obtained from in-solution digestion proteomes.**
(DOCX)

Table S2 **The most important over-represented and under-represented markers responsible for the clustering obtained from in-gel digestion proteomes.**
(DOCX)

Table S3 **The significant cellular components with related proteins and p-values for under-represented markers.**
(DOCX)

Table S4 **The significant molecular functions with related proteins and p-values for under-represented markers.**
(DOCX)

Table S5 **The significant pathways enriched against KEGG database with related proteins and p-values for under-represented markers.**
(DOCX)

Table S6 **Master molecules obtained from upstream regulator analysis for under-represented proteins.**
(DOCX)

Table S7 **The significant biological processes with related proteins and p-values for over-represented markers.**
(DOCX)

Table S8 **The significant cellular components with related proteins and p-values for over-represented markers.**
(DOCX)

Table S9 **The significant molecular functions with related proteins and p-values for over-represented markers.**
(XLSX)

Table S10 **Master molecules obtained from upstream regulator analysis for over-represented proteins.**
(DOCX)
