# Supplementary material for: Urinary Prognostic Biomarkers and Classification of IgA Nephropathy by High Resolution Mass Spectrometry Coupled with Liquid Chromatography
Source: PLoS One. 2013 Dec 5;8(12):e80830. doi: 10.1371/journal.pone.0080830 (PMC3855054; doi:10.1371/journal.pone.0080830)
Supplement: Table S1 — The most important 18 markers responsible for the clustering obtained from in-solution digestion proteomes. (DOCX) [file pone.0080830.s001.docx]

| Ensembl Gene | SwissProt (name) | Median High stage | Median Low stage | Ratio High/Low | Log2(High/Low) |
| --- | --- | --- | --- | --- | --- |
| ENSG00000189058 | APOD_HUMAN | 2.62E+09 | 8.6E+09 | 0.304 | -1.71753 |
| ENSG00000187681 | A1AG1_HUMAN | 1.41E+11 | 2.82E+11 | 0.498 | -1.00513 |
| ENSG00000160862 | ZA2G_HUMAN | 5.55E+10 | 1.06E+11 | 0.526 | -0.92668 |
| ENSG00000121410 | A1BG_HUMAN | 2.99E+10 | 5.58E+10 | 0.536 | -0.89883 |
| ENSG00000079557 | AFAM_HUMAN | 1.34E+09 | 2.31E+09 | 0.58 | -0.78566 |
| ENSG00000171236 | A2GL_HUMAN | 4.85E+09 | 8.23E+09 | 0.589 | -0.76352 |
| ENSG00000047457 | CERU_HUMAN | 1.39E+10 | 2.32E+10 | 0.598 | -0.74113 |
| ENSG00000106927 | AMBP_HUMAN | 1.47E+11 | 5.65E+10 | 2.59 | 1.374578 |
| ENSG00000145192 | FETUA_HUMAN | 3.28E+10 | 1.08E+10 | 3.05 | 1.609359 |
| ENSG00000122194 | PLMN_HUMAN | 1.7E+10 | 5.41E+09 | 3.15 | 1.653168 |
| ENSG00000117601 | ANT3_HUMAN | 6.01E+09 | 1.14E+09 | 5.3 | 2.404673 |
| ENSG00000110169 | HEMO_HUMAN | 7.41E+09 | 1.24E+09 | 5.97 | 2.57816 |
| ENSG00000118137 | APOA1_HUMAN | 1.07E+10 | 1.47E+09 | 7.27 | 2.861881 |
| ENSG00000125730 | CO3_HUMAN | 9.01E+09 | 1.1E+09 | 8.23 | 3.040596 |
| ENSG00000145321 | VTDB_HUMAN | 2.24E+10 | 2.02E+09 | 11.1 | 3.471423 |
| ENSG00000215754 | APOA4_HUMAN | 5.98E+09 | 5.1E+08 | 11.7 | 3.551576 |
| ENSG00000166710 | B2MG_HUMAN | 4.36E+09 | 2.64E+08 | 16.5 | 4.044063 |
| ENSG00000138207 | RET4_HUMAN | 1.14E+10 | 3.23E+08 | 35.3 | 5.141356 |
