# Supplementary material for: Urinary Prognostic Biomarkers and Classification of IgA Nephropathy by High Resolution Mass Spectrometry Coupled with Liquid Chromatography
Source: PLoS One. 2013 Dec 5;8(12):e80830. doi: 10.1371/journal.pone.0080830 (PMC3855054; doi:10.1371/journal.pone.0080830)
Supplement: Table S2 — The most important over-represented and under-represented markers responsible for the clustering obtained from in-gel digestion proteomes. (DOCX) [file pone.0080830.s002.docx]

| PROTEIN ID (underrepresented) | median high | median low | ratio(high/low) | ratio low/high | log(high/low) | PROTEIN ID (overrepresented) | median high | median low | ratio(high/low) | ratio low/high | log(high/low) |
| --- | --- | --- | --- | --- | --- | --- | --- | --- | --- | --- | --- |
| PEPA5 | 1.35E+08 | 8.97E+08 | 0.150357 | 6.650826 | -2.73353 | LV109 | 5.39E+09 | 3.38E+09 | 1.595863 | 0.62662 | 0.674337 |
| DNAS1 | 51527156 | 3.28E+08 | 0.157295 | 6.357494 | -2.66846 | REVERSED-WNK1 | 2.23E+10 | 1.37E+10 | 1.627869 | 0.6143 | 0.702984 |
| EGF | 3.78E+08 | 2.05E+09 | 0.184367 | 5.423957 | -2.43935 | CO3 | 1.97E+10 | 1.12E+10 | 1.755508 | 0.569636 | 0.811889 |
| ATRN | 83870069 | 4.48E+08 | 0.187317 | 5.33855 | -2.41645 | IGLL5 | 8.76E+10 | 4.99E+10 | 1.755935 | 0.569497 | 0.812239 |
| BST1 | 45924491 | 1.96E+08 | 0.23444 | 4.265478 | -2.09271 | A2MG | 7.23E+09 | 3.92E+09 | 1.843578 | 0.542424 | 0.882508 |
| AMPN | 86908400 | 3.6E+08 | 0.24126 | 4.1449 | -2.05134 | AMBP | 5.63E+11 | 2.84E+11 | 1.985301 | 0.503702 | 0.989357 |
| DEFB1 | 43962418 | 1.79E+08 | 0.245826 | 4.067915 | -2.02429 | IGHG2 | 4.49E+11 | 2.09E+11 | 2.150047 | 0.465106 | 1.104368 |
| CD44 | 3.85E+08 | 1.43E+09 | 0.269757 | 3.707041 | -1.89027 | PROF1 | 1.76E+08 | 80003570 | 2.198829 | 0.454788 | 1.136735 |
| TITIN | 2.09E+09 | 7.43E+09 | 0.281648 | 3.550533 | -1.82804 | HV206 | 9.05E+08 | 4.09E+08 | 2.213359 | 0.451802 | 1.146237 |
| DPP4 | 2.28E+08 | 8.07E+08 | 0.282402 | 3.541051 | -1.82418 | A2AP | 2.4E+08 | 1.02E+08 | 2.346117 | 0.426236 | 1.230275 |
| LAMP2 | 1.45E+08 | 5E+08 | 0.289618 | 3.452828 | -1.78778 | CO4B | 4.61E+09 | 1.94E+09 | 2.382658 | 0.419699 | 1.252572 |
| DIAC | 2.02E+08 | 5.94E+08 | 0.340838 | 2.933943 | -1.55284 | HPT | 1.57E+10 | 6.44E+09 | 2.443827 | 0.409194 | 1.289142 |
| S10A6 | 23067044 | 63512958 | 0.363186 | 2.753407 | -1.46122 | APOA4 | 7.85E+09 | 2.96E+09 | 2.648591 | 0.377559 | 1.405225 |
| CD59 | 7.91E+09 | 2.17E+10 | 0.364741 | 2.741672 | -1.45506 | UTER | 2.04E+10 | 7.58E+09 | 2.686174 | 0.372277 | 1.425553 |
| FINC | 30746313 | 79675956 | 0.385892 | 2.591399 | -1.37373 | K1C17 | 6.85E+09 | 2.52E+09 | 2.718897 | 0.367796 | 1.443022 |
| THBG | 6.44E+09 | 1.64E+10 | 0.391916 | 2.551566 | -1.35138 | HEMO | 1.24E+10 | 4.49E+09 | 2.767454 | 0.361343 | 1.468559 |
| OSTP | 9.26E+08 | 2.36E+09 | 0.392344 | 2.548781 | -1.34981 | ITIH2 | 8.63E+08 | 2.94E+08 | 2.932764 | 0.340975 | 1.552261 |
| VMO1 | 2.54E+08 | 6.42E+08 | 0.394928 | 2.532105 | -1.34034 | FFAR2 | 6.26E+08 | 2E+08 | 3.128004 | 0.319693 | 1.645242 |
| UROM | 7.46E+09 | 1.88E+10 | 0.396061 | 2.52486 | -1.3362 | VTNC | 6.99E+08 | 1.94E+08 | 3.608752 | 0.277104 | 1.8515 |
| GNS | 1.01E+09 | 2.5E+09 | 0.402438 | 2.484856 | -1.31316 | LYSC | 2.36E+09 | 6.16E+08 | 3.840065 | 0.260412 | 1.941131 |
| ASAH1 | 2.37E+08 | 5.46E+08 | 0.433337 | 2.307671 | -1.20644 | HV107 | 4.71E+09 | 1.21E+09 | 3.895461 | 0.256709 | 1.961794 |
| RNAS2 | 4.48E+09 | 1.02E+10 | 0.437841 | 2.283933 | -1.19152 | TTHY | 5.84E+10 | 1.32E+10 | 4.424057 | 0.226037 | 2.14537 |
| LAIR1 | 3.24E+08 | 6.87E+08 | 0.47143 | 2.121204 | -1.08488 | VTDB | 5.58E+10 | 1.05E+10 | 5.323008 | 0.187864 | 2.412242 |
| CATB | 1.23E+08 | 2.52E+08 | 0.487937 | 2.049446 | -1.03523 | APOA1 | 2.11E+10 | 3.6E+09 | 5.874657 | 0.170223 | 2.554505 |
| A2GL | 2.18E+10 | 4.47E+10 | 0.488169 | 2.048471 | -1.03455 | ANGT | 7.37E+09 | 1.09E+09 | 6.745162 | 0.148254 | 2.753853 |
| BTD | 2.5E+08 | 4.9E+08 | 0.510174 | 1.960117 | -0.97094 | CFAB | 4.7E+08 | 68181385 | 6.893104 | 0.145073 | 2.785154 |
| AMY2B | 1.69E+09 | 3.23E+09 | 0.523237 | 1.911178 | -0.93446 | GUC2A | 5.24E+08 | 57739736 | 9.07237 | 0.110225 | 3.181479 |
| SAP3 | 5.62E+09 | 1.02E+10 | 0.549212 | 1.820789 | -0.86456 | CYTM | 9.11E+09 | 9.54E+08 | 9.543876 | 0.104779 | 3.254575 |
| AFAM | 1.13E+10 | 2.03E+10 | 0.556048 | 1.798405 | -0.84672 | CYTC | 4.91E+09 | 5.08E+08 | 9.660014 | 0.10352 | 3.272025 |
| VASN | 1.26E+09 | 2.23E+09 | 0.563355 | 1.775081 | -0.82788 | B2MG | 2.54E+11 | 8.09E+09 | 31.4081 | 0.031839 | 4.973065 |
| CERU | 6.16E+10 | 1.06E+11 | 0.583239 | 1.714562 | -0.77784 | RET4 | 7.53E+10 | 1.44E+09 | 52.3335 | 0.019108 | 5.709663 |
