# Supplementary material for: Urinary Prognostic Biomarkers and Classification of IgA Nephropathy by High Resolution Mass Spectrometry Coupled with Liquid Chromatography
Source: PLoS One. 2013 Dec 5;8(12):e80830. doi: 10.1371/journal.pone.0080830 (PMC3855054; doi:10.1371/journal.pone.0080830)
Supplement: Table S3 — The significant cellular components with related proteins and p-values for under-represented markers. (DOCX) [file pone.0080830.s003.docx]

| Peotein Namne | Cellular Component | p- value | Enrichment score |
| --- | --- | --- | --- |
| AMPN | soluble fraction | 0.04 | 0.78 |
| CATB | soluble fraction | 0.04 | 0.78 |
| DPP4 | soluble fraction | 0.04 | 0.78 |
| EGF | soluble fraction | 0.04 | 0.78 |
| AMPN | vesicle lumen | 0.006 | 1.15 |
| EGF | vesicle lumen | 0.006 | 1.15 |
| FINC | vesicle lumen | 0.006 | 1.15 |
| AMPN | memberane-bounded vesicle | 0.014 | 1.15 |
| CATB | memberane-bounded vesicle | 0.014 | 1.15 |
| EGF | memberane-bounded vesicle | 0.014 | 1.15 |
| FINC | memberane-bounded vesicle | 0.014 | 1.15 |
| LAMP2 | memberane-bounded vesicle | 0.014 | 1.15 |
| OSTP | memberane-bounded vesicle | 0.014 | 1.15 |
| CD59 | anchored to memberane | 0.019 | 1.78 |
| BST1 | anchored to memberane | 0.019 | 1.78 |
| CERU | anchored to memberane | 0.019 | 1.78 |
| UROM | anchored to memberane | 0.019 | 1.78 |
| CD44 | apical part of plasma memberane | 0.0036 | 3.53 |
| CATB | apical part of plasma memberane | 0.0036 | 3.53 |
| DPP4 | apical part of plasma memberane | 0.0036 | 3.53 |
| FINC | apical part of plasma memberane | 0.0036 | 3.53 |
| UROM | apical part of plasma memberane | 0.0036 | 3.53 |
| SAP3 | lysosome | 0.00001 | 4.34 |
| ASAH1 | lysosome | 0.00001 | 4.34 |
| CATB | lysosome | 0.00001 | 4.34 |
| DIAC | lysosome | 0.00001 | 4.34 |
| GNS | lysosome | 0.00001 | 4.34 |
| LAMP2 | lysosome | 0.00001 | 4.34 |
| RNAS2 | lysosome | 0.00001 | 4.34 |
