# Supplementary material for: Urinary Prognostic Biomarkers and Classification of IgA Nephropathy by High Resolution Mass Spectrometry Coupled with Liquid Chromatography
Source: PLoS One. 2013 Dec 5;8(12):e80830. doi: 10.1371/journal.pone.0080830 (PMC3855054; doi:10.1371/journal.pone.0080830)
Supplement: Table S4 — The significant molecular functions with related proteins and p-values for under-represented markers. (DOCX) [file pone.0080830.s004.docx]

| Peotein Namne | Molecular Function | p- value | Enrichment score |
| --- | --- | --- | --- |
| CD44 | collagen binding | 0.002 | 3.53 |
| DPP4 | collagen binding | 0.002 | 3.53 |
| FINC | collagen binding | 0.002 | 3.53 |
| AMPN | peptide binding | 0.054 | 3.53 |
| CATB | peptide binding | 0.054 | 3.53 |
| DPP4 | peptide binding | 0.054 | 3.53 |
