# Supplementary material for: Urinary Prognostic Biomarkers and Classification of IgA Nephropathy by High Resolution Mass Spectrometry Coupled with Liquid Chromatography
Source: PLoS One. 2013 Dec 5;8(12):e80830. doi: 10.1371/journal.pone.0080830 (PMC3855054; doi:10.1371/journal.pone.0080830)
Supplement: Table S5 — The significant pathways enriched against KEGG database with related proteins and p-values for under-represented markers. (DOCX) [file pone.0080830.s005.docx]

| Protein Name | Name of pathway | p-value | Enrichment score |
| --- | --- | --- | --- |
| CD44 | ECM-receptor interaction | 0.02 | 2.33 |
| FINC | ECM-receptor interaction | 0.02 | 2.33 |
| OSTP | ECM-receptor interaction | 0.02 | 2.33 |
| SAP3 | lysosome | 0.0003 | 4.34 |
| ASAH1 | lysosome | 0.0003 | 4.34 |
| CATB | lysosome | 0.0003 | 4.34 |
| GNS | lysosome | 0.0003 | 4.34 |
| LAMP2 | lysosome | 0.0003 | 4.34 |
