# Supplementary material for: Urinary Prognostic Biomarkers and Classification of IgA Nephropathy by High Resolution Mass Spectrometry Coupled with Liquid Chromatography
Source: PLoS One. 2013 Dec 5;8(12):e80830. doi: 10.1371/journal.pone.0080830 (PMC3855054; doi:10.1371/journal.pone.0080830)
Supplement: Table S6 — Master molecules obtained from upstream regulator analysis for under-represented proteins. (DOCX) [file pone.0080830.s006.docx]

| ID | Master molecule name | Maximal radius | Reached from set | Reachable total | Score | FDR | Z-Score | Ranks sum | Hits names |
| --- | --- | --- | --- | --- | --- | --- | --- | --- | --- |
| MO000125588 | Osteopontin-isoform2(h) | 1.20864 | 37 | 556 | 0.909091 | 0.03 | 1.220008 | 44 | ["CD44-CTF(h)","CD44-isoform1(h)","CD44-isoform10(h)","CD44-isoform11(h)","CD44-isoform12(h)","CD44-isoform13(h)","CD44-isoform14(h)","CD44-isoform15(h)","CD44-isoform16(h)","CD44-isoform17(h)","CD44-isoform2(h)","CD44-isoform3(h)","CD44-isoform4(h)","CD44-isoform5(h)","CD44-isoform6(h)","CD44-isoform7(h)","CD44-isoform8(h)","CD44-isoform9(h)","Osteopontin-isoform2(h)","Osteopontin-p45(h)","Osteopontin-p50(h)","fibronectin-1(h)","fibronectin-10(h)","fibronectin-11(h)","fibronectin-12(h)","fibronectin-2(h)","fibronectin-3(h)","fibronectin-4(h)","fibronectin-5(h)","fibronectin-6(h)","fibronectin-7(h)","fibronectin-8(h)","fibronectin-9(h)","fibronectin-isoform13(h)","fibronectin-isoform14(h)","fibronectin-isoform15(h)","fibronectin-xbb13(h)"] |
| MO000179092 | UMOD-isoform1(h) | 1.98618 | 24 | 48 | 0.574267 | 0 | 5.28178 | 22 | ["CD44-isoform1(h)","CD44-isoform10(h)","CD44-isoform11(h)","CD44-isoform12(h)","CD44-isoform13(h)","CD44-isoform14(h)","CD44-isoform15(h)","CD44-isoform16(h)","CD44-isoform17(h)","CD44-isoform2(h)","CD44-isoform3(h)","CD44-isoform4(h)","CD44-isoform5(h)","CD44-isoform6(h)","CD44-isoform7(h)","CD44-isoform8(h)","CD44-isoform9(h)","Osteopontin-isoform1(h)","Osteopontin-isoform2(h)","Osteopontin-isoform3(h)","Osteopontin-isoform4(h)","Osteopontin-p45(h)","Osteopontin-p50(h)","UMOD-isoform1(h)"] |
| MO000083712 | fibronectin-xbb13(h) | 1.83605 | 24 | 237 | 0.404746 | 0.012 | 1.932761 | 35 | ["CD44-isoform1(h)","CD44-isoform10(h)","CD44-isoform11(h)","CD44-isoform12(h)","CD44-isoform13(h)","CD44-isoform14(h)","CD44-isoform15(h)","CD44-isoform16(h)","CD44-isoform17(h)","CD44-isoform2(h)","CD44-isoform3(h)","CD44-isoform4(h)","CD44-isoform5(h)","CD44-isoform6(h)","CD44-isoform7(h)","CD44-isoform8(h)","CD44-isoform9(h)","Osteopontin-isoform1(h)","Osteopontin-isoform2(h)","Osteopontin-isoform3(h)","Osteopontin-isoform4(h)","Osteopontin-p45(h)","Osteopontin-p50(h)","fibronectin-xbb13(h)"] |
| MO000102887 | biotin-holoenzyme | 1.78297 | 1 | 1 | 0.208296 | 0 | 1 | 68 | ["biotinidase(h)"] |
| MO000112176 | adam10(h) | 1.57218 | 3 | 42 | 0.206771 | 0.004 | 4.068008 | 44 | ["EGF(h)","EGF-ECD(h)","EGF-isoform2(h)"] |
| MO000117753 | mmp2(h) | 1.17752 | 16 | 69 | 0.20578 | 0.004 | 3.219638 | 49 | ["fibronectin-1(h)","fibronectin-10(h)","fibronectin-11(h)","fibronectin-12(h)","fibronectin-2(h)","fibronectin-3(h)","fibronectin-4(h)","fibronectin-5(h)","fibronectin-6(h)","fibronectin-7(h)","fibronectin-8(h)","fibronectin-9(h)","fibronectin-isoform13(h)","fibronectin-isoform14(h)","fibronectin-isoform15(h)","fibronectin-xbb13(h)"] |
| MO000109636 | TGC-isoform2(h) | 1.32461 | 16 | 103 | 0.204544 | 0.002 | 2.606231 | 51 | ["fibronectin-1(h)","fibronectin-10(h)","fibronectin-11(h)","fibronectin-12(h)","fibronectin-2(h)","fibronectin-3(h)","fibronectin-4(h)","fibronectin-5(h)","fibronectin-6(h)","fibronectin-7(h)","fibronectin-8(h)","fibronectin-9(h)","fibronectin-isoform13(h)","fibronectin-isoform14(h)","fibronectin-isoform15(h)","fibronectin-xbb13(h)"] |
| MO000117396 | MT1-MMP(h) | 1.63784 | 17 | 33 | 0.176039 | 0.008 | 4.229628 | 48 | ["CD44-isoform1(h)","CD44-isoform10(h)","CD44-isoform11(h)","CD44-isoform12(h)","CD44-isoform13(h)","CD44-isoform14(h)","CD44-isoform15(h)","CD44-isoform16(h)","CD44-isoform17(h)","CD44-isoform2(h)","CD44-isoform3(h)","CD44-isoform4(h)","CD44-isoform5(h)","CD44-isoform6(h)","CD44-isoform7(h)","CD44-isoform8(h)","CD44-isoform9(h)"] |
| MO000037507 | gamma-secretase(h) | 0.998952 | 3 | 52 | 0.086092 | 0.022 | 3.923271 | 52 | ["CD44-CTF(h)","CD44-ICD(h)","CD44-beta(h)"] |
| MO000038019 | CD44-CTF(h) | 1.00893 | 3 | 3 | 0.04974 | 0 | 15.71227 | 31 | ["CD44-CTF(h)","CD44-ICD(h)","CD44-beta(h)"] |
