# Supplementary material for: Urinary Prognostic Biomarkers and Classification of IgA Nephropathy by High Resolution Mass Spectrometry Coupled with Liquid Chromatography
Source: PLoS One. 2013 Dec 5;8(12):e80830. doi: 10.1371/journal.pone.0080830 (PMC3855054; doi:10.1371/journal.pone.0080830)
Supplement: Table S7 — The significant biological processes with related proteins and p-values for over-represented markers. (DOCX) [file pone.0080830.s007.docx]

| Protein Name | Biological process | p-value | Enrichment score |
| --- | --- | --- | --- |
| A2MG | acute inflammatory response | 2.10E-05 | 4.06 |
| CO4B | acute inflammatory response | 2.10E-05 | 4.06 |
| CFAB | acute inflammatory response | 2.10E-05 | 4.06 |
| A2AP | acute inflammatory response | 2.10E-05 | 4.06 |
| CO3 | acute inflammatory response | 2.10E-05 | 4.06 |
| ANGT | regulation of cholesterol esterification | 7.70E-05 | 2.09 |
| APOA1 | regulation of cholesterol esterification | 7.70E-05 | 2.09 |
| APOA4 | regulation of cholesterol esterification | 7.70E-05 | 2.09 |
| B2MG | positive regulation of immune response | 9.60E-05 | 3.19 |
| CO4B | positive regulation of immune response | 9.60E-05 | 3.19 |
| CFAB | positive regulation of immune response | 9.60E-05 | 3.19 |
| HEMO | positive regulation of immune response | 9.60E-05 | 3.19 |
| CO3 | positive regulation of immune response | 9.60E-05 | 3.19 |
| ANGT | homeostatic process | 7.60E-03 | 1.46 |
| APOA1 | homeostatic process | 7.60E-03 | 1.46 |
| APOA4 | homeostatic process | 7.60E-03 | 1.46 |
| HPT | homeostatic process | 7.60E-03 | 1.46 |
| HEMO | homeostatic process | 7.60E-03 | 1.46 |
| RET4 | homeostatic process | 7.60E-03 | 1.46 |
| ANGT | hormone metabolic process | 1.40E-02 | 2.45 |
| APOA1 | hormone metabolic process | 1.40E-02 | 2.45 |
| RET4 | hormone metabolic process | 1.40E-02 | 2.45 |
| ANGT | regulation of protein amino acid phosphorylation | 3.50E-02 | 1.46 |
| APOA1 | regulation of protein amino acid phosphorylation | 3.50E-02 | 1.46 |
| HEMO | regulation of protein amino acid phosphorylation | 3.50E-02 | 1.46 |
| A2MG | response to nutrient levels | 4.40E-02 | 1.44 |
| CFAB | response to nutrient levels | 4.40E-02 | 1.44 |
| RET4 | response to nutrient levels | 4.40E-02 | 1.44 |
