# Supplementary material for: Urinary Prognostic Biomarkers and Classification of IgA Nephropathy by High Resolution Mass Spectrometry Coupled with Liquid Chromatography
Source: PLoS One. 2013 Dec 5;8(12):e80830. doi: 10.1371/journal.pone.0080830 (PMC3855054; doi:10.1371/journal.pone.0080830)
Supplement: Table S8 — The significant cellular components with related proteins and p-values for over-represented markers. (DOCX) [file pone.0080830.s008.docx]

| Protein Name | Cellular component | p-value | Enrichment score |
| --- | --- | --- | --- |
| AMBP | extra cellular region | 1.40E-17 | 15.31 |
| FETUA | extra cellular region | 1.40E-17 | 15.31 |
| A2MG | extra cellular region | 1.40E-17 | 15.31 |
| ANGT | extra cellular region | 1.40E-17 | 15.31 |
| APOA1 | extra cellular region | 1.40E-17 | 15.31 |
| APOA4 | extra cellular region | 1.40E-17 | 15.31 |
| B2MG | extra cellular region | 1.40E-17 | 15.31 |
| CO4B | extra cellular region | 1.40E-17 | 15.31 |
| CFAB | extra cellular region | 1.40E-17 | 15.31 |
| CYTC | extra cellular region | 1.40E-17 | 15.31 |
| CYTM | extra cellular region | 1.40E-17 | 15.31 |
| VTDB | extra cellular region | 1.40E-17 | 15.31 |
| GUC2A | extra cellular region | 1.40E-17 | 15.31 |
| HPT | extra cellular region | 1.40E-17 | 15.31 |
| HEMO | extra cellular region | 1.40E-17 | 15.31 |
| IGHG2 | extra cellular region | 1.40E-17 | 15.31 |
| ITIH2 | extra cellular region | 1.40E-17 | 15.31 |
| LYSC | extra cellular region | 1.40E-17 | 15.31 |
| PLMN | extra cellular region | 1.40E-17 | 15.31 |
| RET4 | extra cellular region | 1.40E-17 | 15.31 |
| UTER | extra cellular region | 1.40E-17 | 15.31 |
| ANT3 | extra cellular region | 1.40E-17 | 15.31 |
| A2AP | extra cellular region | 1.40E-17 | 15.31 |
| CO3 | extra cellular region | 1.40E-17 | 15.31 |
| TTHY | extra cellular region | 1.40E-17 | 15.31 |
| VTNC | extra cellular region | 1.40E-17 | 15.31 |
| APOA1 | plasma lipoprotein particle | 2.60E-03 | 2.64 |
| APOA4 | plasma lipoprotein particle | 2.60E-03 | 2.64 |
| HPT | plasma lipoprotein particle | 2.60E-03 | 2.64 |
