# Supplementary material for: Urinary Prognostic Biomarkers and Classification of IgA Nephropathy by High Resolution Mass Spectrometry Coupled with Liquid Chromatography
Source: PLoS One. 2013 Dec 5;8(12):e80830. doi: 10.1371/journal.pone.0080830 (PMC3855054; doi:10.1371/journal.pone.0080830)
Supplement: Table S10 — Master molecules obtained from upstream regulator analysis for over-represented proteins. (DOCX) [file pone.0080830.s010.docx]

| ID | Master molecule name | Maximal radius | Reached from set | Reachable total | Score | FDR | Z-Score | Ranks sum | Hits names |
| --- | --- | --- | --- | --- | --- | --- | --- | --- | --- |
| MO000169289 | trypsin-1(h) | 1.575 | 3 | 10 | 0.087354 | 0 | 11.06495 | 12 | ["C3a(h)","C3b(h)","complement C3(h)"] |
| MO000126830 | ADAM19-isoform2(h) | 1.575 | 1 | 1 | 0.160032 | 0 | 22.367 | 9 | ["alpha2M(h)"] |
| MO000056756 | PTP1B(h) | 1.575 | 1 | 80 | 0.320399 | 0 | 4.317034 | 12 | ["Lysozyme C(h)"] |
| MO000168388 | CD45-isoform2(h) | 1.575 | 1 | 64 | 0.326332 | 0 | 4.403864 | 9 | ["Lysozyme C(h)"] |
| MO000168387 | CD45-isoform1(h) | 1.575 | 1 | 64 | 0.326332 | 0 | 4.316281 | 12 | ["Lysozyme C(h)"] |
| MO000102727 | myeloperoxidase isoform-H7(h) | 1.9 | 1 | 5 | 0.460925 | 0 | 1 | 9 | ["Apo-AI(h)"] |
| MO000102725 | myeloperoxidase isoform-H17(h) | 1.9 | 1 | 5 | 0.460925 | 0 | 1 | 11 | ["Apo-AI(h)"] |
| MO000102726 | myeloperoxidase isoform-H14(h) | 1.9 | 1 | 5 | 0.460925 | 0 | 1 | 15 | ["Apo-AI(h)"] |
| MO000126242 | IF(h) | 1 | 1 | 6 | 0.483808 | 0 | 15.79196 | 3 | ["complement C3(h)"] |
| MO000162028 | renin-isoform1(h) | 1.8 | 1 | 1 | 0.499376 | 0 | 1 | 8 | ["angiotensinogen(h)"] |
| MO000162030 | renin-isoform2(h) | 1.8 | 1 | 1 | 0.499376 | 0 | 1 | 10 | ["angiotensinogen(h)"] |
